# Metastatic small intestinal neuroendocrine tumor presented with partial intestinal obstruction in Saudi Arabia: a case report

**DOI:** 10.1093/jscr/rjaf307

**Published:** 2025-05-17

**Authors:** Faris Alsobyani, Mansour Almalki, Hassan Abu Rokbah

**Affiliations:** General Surgery Department, Al Noor Specialist Hospital, 3rd Ring Rd, 24241, Makkah, Saudi Arabia; General Surgery Department, Al Noor Specialist Hospital, 3rd Ring Rd, 24241, Makkah, Saudi Arabia; General Surgery Department, Al Noor Specialist Hospital, 3rd Ring Rd, 24241, Makkah, Saudi Arabia

**Keywords:** neuroendocrine tumors, ileocolic neoplasms, incidental findings, bowel obstruction

## Abstract

Well-differentiated neuroendocrine tumors (NETs) of the ileocecal region are rare but increasingly recognized gastrointestinal neoplasms. They often present vague symptoms, delaying diagnosis. Despite slow growth, these tumors can metastasize to the liver and bone, complicating management. Despite their indolent nature, these tumors can metastasize to the liver and bone, complicating treatment. We report a case of a 54-year-old woman with a one-year history of vague abdominal symptoms that worsened over the last 2 months. Contrast-enhanced computed tomography imaging revealed a mesenteric lesion leading to partial intestinal obstruction. Further imaging revealed liver and spine metastases. Colonoscopy confirmed an obstructing ileocecal mass, and biopsy identified a Grade 1 well-differentiated NET. The patient underwent laparoscopic right hemicolectomy with ileocolic anastomosis for symptom relief, followed by octreotide therapy. This case highlights the diagnostic and therapeutic challenges of metastatic ileocecal NETs and emphasizes the importance of a multidisciplinary approach for effective treatment and long-term stability.

## Introduction

Well-differentiated neuroendocrine tumors (NETs) of the ileocecal region are rare gastrointestinal neoplasms with increasing incidence. Small intestinal NETs are among the most common, particularly in the ileum [1]. These tumors often present nonspecific symptoms, delaying diagnosis, with less than a third carcinoid syndrome [2]. While low-grade NETs exhibit slow proliferation, they frequently metastasize to the liver and occasionally involve bone [3, 4]. Effective management requires a combination of surgery and medical therapy, such as somatostatin analogs [5]. Data on the presentation and management of metastatic NETs from the Middle East, particularly Saudi Arabia, remain limited. We provide in this report a case of ileocolic mass with partial bowel obstruction in Saudi Arabia.

## Case report

**Figure 1:**
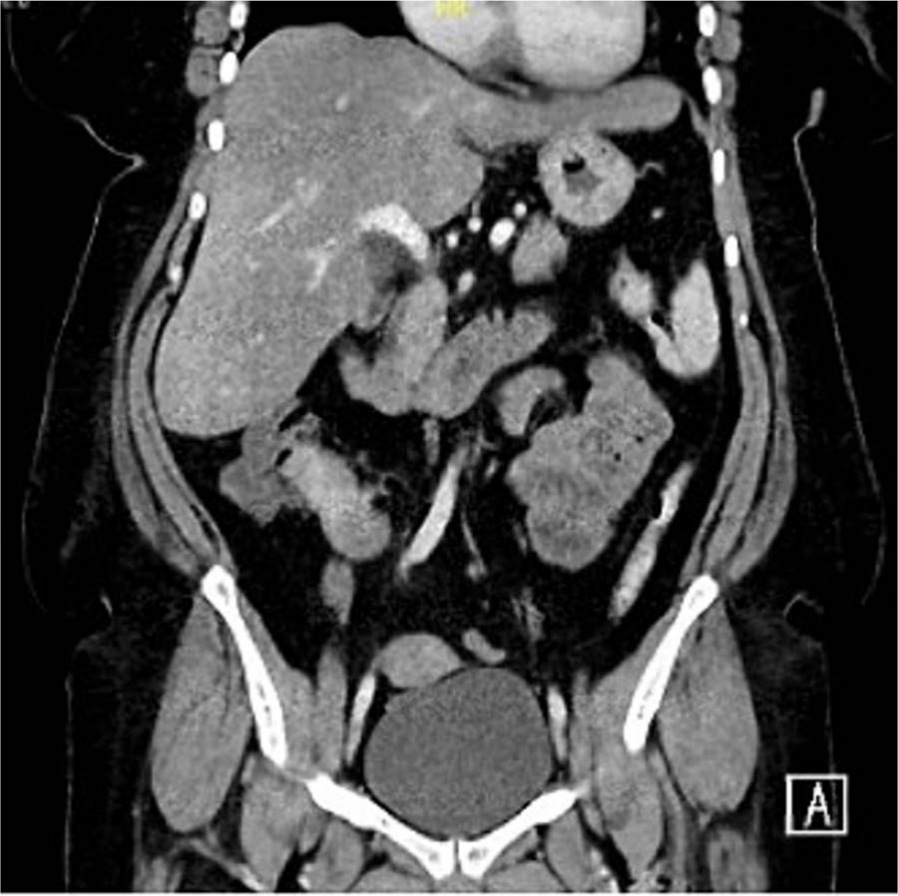
Hyper-enhancing spiculated ill-defined lesion at the right lower mesentery measured about 2.9 × 2.2 × 2.4 cm, with no internal calcification or macroscopic component associated with thickening and tethering of the adjacent bowel loops.

A 54-year-old female presented with intermittent right lower quadrant abdominal pain, distension, and constipation persisting for 1 year, with symptom progression in the last 2 months. There are no other gastrointestinal nor constitutional symptoms. The patient has no previous medical or surgical history. On examination, the patient was alert, oriented, and hemodynamically stable. The abdomen was mildly distended, however, soft, with right lower quadrant mild tenderness and localized fullness. A digital rectal examination revealed an empty rectum with no visible or palpable pathology. Laboratory workup was insignificant. Erect and supine abdominal X-rays, performed to evaluate possible obstruction, showed no abnormalities. Computed tomography (CT) scan (Fig. 1) showed a hyper-enhancing spiculated ill-defined lesion at the right lower mesentery measuring about 2.9 × 2.2 × 2.4 cm, with no internal calcification or macroscopic component associated with thickening and tethering of the adjacent bowel loops named terminal ileum and cecum, causing tethering and stranding of the mesentery. No mechanical bowel obstruction.

The decision was taken to admit this patient as a case of a partially obstructing small bowel tumor for further workup and management. Tumor markers were negative. CT chest, abdomen, and pelvis revealed liver metastasis (segment VIII) and spine metastasis (T8). A colonoscopy revealed an obstructing ileocecal mass, from which biopsies were taken, and demonstrating a well-differentiated NET (G1).

The case was discussed on a multidisciplinary team meeting, and a decision was made in favor of operative management in order to relieve the obstruction despite the presence of metastasis. She underwent a laparoscopic right hemicolectomy and ileocolic anastomosis without any immediate complications. On histopathology examination, a well-differentiated NET (G1) was seen. A few days later, the patient was discharged home in a good condition on daily octreotide. Follow-up in the outpatient department for 33 months showed a good status and quality of life with no complaints.

## Discussion

Well-differentiated NETs of the ileocecal region represent a distinct subset of gastrointestinal neoplasms. Recent epidemiological data indicate an increasing incidence of gastrointestinal NETs, with small bowel tumors, particularly in the ileum, being among the most prevalent. A study by Dasari *et al*. reported that the annual incidence of NETs has risen over the past few decades, with small intestinal NETs constituting a significant proportion of these cases [1]. These tumors are often diagnosed in individuals aged between 50 and 70 years, with a slight female predominance, aligning with our patient’s profile of a 54-year-old female.

The clinical features of NETs are somewhat unique because symptoms assigned to NETs may vary from patient to patient [6]. Our patient complained of mild and diffuse abdominal discomfort, distension, and constipation, which he developed one year before admission, which worsened in the last 2 months. The nonspecific nature of these symptoms frequently leads to misdiagnosis and delays in appropriate management. Most importantly, the patient does not present with classic carcinoid syndrome symptoms such as flushing, diarrhea, and wheezing, which are often observed in cases where carcinoid tumors cause liver metastases and substances responsible for these symptoms are not metabolized by the liver. Modlin *et al*. also noted that less than a third of patients with NETs develop carcinoid syndrome, explaining why the tumor is rather difficult to diagnose [2].

From a pathophysiologic perspective, well-differentiated NETs grow slowly and tend to metastasize. The Ki-67 proliferation index is a critical marker for tumor grading and prognostication. The tumor was categorized as Grade 1 (G1); the Ki-67 of the patient was less than 3%; hence, indicating low proliferative potential. But even with such a low index, a CT scan detected liver and spine metastases. This is confirmed by Yao *et al*. who noted that even low-grade NETs could metastasize most commonly to the liver, which is among the most frequent distant sites involved [3].

A number of diagnostic techniques are required in order to diagnose NETs. An abdominal X-ray obtained in our patient upon admission was normal, and early NETs are often missed, as seen in this case. However, contrast-enhanced CT images obtained revealed a mass in the terminal ileum with a feature of partial intestinal obstruction. Serum and urine chromogranin A and 5-HIAA levels were within normal limits, reflecting the limited sensitivity of these markers in non-secretory NETs. An ileocecal mass that was obstructing the colon was diagnosed through colonoscopy by direct visualization and biopsy. CT of the chest, abdomen, and pelvis with advanced imaging provided better characterization of the metastatic disease involving the liver and spine. The results of this study are consistent with the diagnostic procedures and imaging techniques described by Öberg *et al*., where the authors established that tumor markers are far from perfect and imaging is paramount in the identification of NETs [7].

The liver is generally the most frequent site for metastatic spread in NETs; however, bone metastases, though less common, can be of substantial clinical concern. This kind of metastasis in the liver and spine shows that NETs present an unpredictable configuration of metastases. As reported by Strosberg *et al*., liver metastases are observed in roughly half the patients at initial diagnosis, and, despite being less frequent than liver involvement, bone metastases are considered to portend a worse outcome [4].

There are several complex therapeutic approaches to NETs. Surgical resection remains the mainstay of treatment, including in previously metastasized cases, especially in the face of the symptoms or structural obstruction. After diagnosis, our patient required a laparoscopic right hemicolectomy for relief of the obstructive process, and an octreotide was started. This approach in management is in agreement with Pape *et al*., who also recommend surgical management in localized and regional stages of the disease in order to enhance survival figures [8]. Also, somatostatin analogs such as octreotide possess symptomatic relief and may slow tumor growth, as pointed out by Rinke *et al*. [5]. Continuing with the operation in the presence of metastases is viewed in concordance with the present guidelines, which indicate that surgery of the primary tumor can be helpful for relieving symptoms and improving the responsiveness of subsequent treatments.

In conclusion, this case illustrates challenges associated with the diagnosis and management of well-differentiated NETs of the ileocecal region when metastatic. This calls for a broad and conservative management plan consisting of surgical and medical treatments.

Targeting every single patient depending on his or her condition. Current research and clinic trials help to modify the understanding and management of this typical and complex group of neoplasms.
